# Effect of evolocumab on the gut microbiota in patients with acute myocardial infarction

**DOI:** 10.1099/jmm.0.002114

**Published:** 2026-01-13

**Authors:** Jie Zou, Yunzhu Peng, Hongyan Cai, Qinghua Zhong, Na Zhu, Wenyi Gu, Fazhi Yang, Tao Shi, Sirui Yang, Lixing Chen

**Affiliations:** 1 Department of Cardiology, Kunming Medical University First Affiliated Hospital, Kunming, Yunnan, PR China; 2 Department of Paediatrics, Kunming Medical University First Affiliated Hospital, Kunming, Yunnan, PR China

**Keywords:** 16S rRNA sequencing, acute myocardial infarction, evolocumab, gut microbiota, outcome

## Abstract

**Introduction.** Growing evidence indicates significant interactions between the intestinal microflora and drugs commonly used to treat coronary heart disease.

**Hypothesis/Gap Statement.** Despite this, research specifically investigating the relationship between proprotein convertase subtilisin/kexin type 9 inhibitors and alterations in the gut microbiota has not been previously published.

**Aim.** This study aimed to identify changes in the gut microbiota potentially associated with evolocumab use in patients with acute myocardial infarction (AMI).

**Methodology.** In this prospective study, 26 AMI patients receiving statins (≥8 weeks) were administered evolocumab (420 mg/4 weeks) alongside standard therapy. Eighteen age-matched healthy volunteers served as controls. 16S rRNA sequencing (NCBI SRA: PRJNA1154993) was subsequently performed on samples from these groups to analyse the gut microbiota community.

**Results.** No significant α-diversity differences were observed among groups (*P*>0.05). *Firmicutes* dominated AMI-evolocumab baseline (0 W: 76.32%) versus post-treatment (8 W: 65.22%) and controls (60.40%), while *Bacteroidota* increased post-treatment (0 W: 15.07%→8 W: 19.99%; control: 27.11%). The second most abundant phyla were *Proteobacteria* and *Actinobacteria*. In addition, the differences in the microbial structure among the three groups were as follows: at the genus level, the results of the genus difference analysis revealed significant differences in the abundances of nine types of bacteria among the three groups. Compared with those in the AMI-evolocumab (0 W) group, the abundances of beneficial bacteria, such as *Odoribacter* and *Parabacteroides*, were increased in the AMI-evolocumab (8 W) group.

**Conclusion.** Our research showed that evolocumab can regulate the gut microbiota of patients with AMI to promote a healthier state, which is beneficial for patients with AMI.

## Data Summary

The data presented in the study are deposited in the NCBI Sequence Read Archive database repository accession number PRJNA1154993. Due to the large number of samples, the complete list of individual dataset accession numbers is provided in Table S1 (available in the online Supplementary Material). The other datasets used and/or analysed during the current study are available from the corresponding author on reasonable request.

## Introduction

Acute myocardial infarction (AMI), characterized by myocardial necrosis resulting from prolonged coronary artery ischaemia, represents a critical manifestation of atherosclerotic cardiovascular disease. Globally, cardiovascular diseases account for 31% of all mortality, with AMI contributing to over 15% of these deaths. Notably, the age-standardized incidence rate of AMI has risen by 18.7% in developing countries during the past decade, paralleling dietary pattern transitions and population ageing [1].

The intestinal microecology system consists of the normal intestinal flora and the environment in which they live. One of the most distinctive features of the intestinal flora is its stability, and if the flora is out of balance, various diseases can occur [2]. In recent years, with the discovery of the liver–intestinal axis, brain–intestinal axis and heart–intestinal axis, researchers have gradually realized that the gut flora can affect other important organs from a distance [3]. The role of gut dysbiosis in coronary heart disease has garnered substantial interest, and it has been shown that the gut flora exhibits characteristic changes with the severity of coronary artery disease (CAD) [4]. The gut microbiota contributes to AMI development via metabolite-dependent pathways. Trimethylamine N-oxide (TMAO), a gut microbiota-derived metabolite from dietary choline and carnitine, promotes endothelial dysfunction and foam cell formation by upregulating scavenger receptors and inhibiting reverse cholesterol transport. Clinical studies demonstrate that elevated serum TMAO levels correlate with a 2.5-fold increased risk of major adverse cardiovascular events in AMI patients. Concurrently, dysbiosis-induced intestinal barrier disruption facilitates bacterial translocation, evidenced by elevated zonulin and LPS in STEMI patients, which exacerbates systemic inflammation and microvascular obstruction [5]. LPS activates Toll-like receptor 4 signalling, amplifying pro-inflammatory cytokines and oxidative stress, thereby accelerating plaque rupture [6]. Multi-omics approaches have identified gut microbiota-derived biomarkers for AMI risk stratification. A cohort study integrating 16S rRNA sequencing and metabolomics revealed distinct microbial signatures in AMI patients, including enriched *Alistipes* and *Streptococcus*, alongside depleted *Roseburia*. These taxa, combined with faecal metabolites, for discriminating AMI from stable CAD, outperform traditional biomarkers like C-reactive Protein [7].

Macrophage-mediated phagocytosis of oxidized low-density lipoprotein cholesterol (LDL-C) and secretion of proinflammatory cytokines critically drive foam cell formation and atherogenesis. The central pathophysiological mechanism of AMI stems from atherosclerotic plaque destabilization and rupture. Crucially, clinical evidence demonstrates that each 1 mmol l^−1^ reduction in LDL-C correlates with a 20–25% decreased risk of recurrent cardiovascular events, including reinfarction and cardiovascular mortality [8]. Currently, domestic guidelines for patients with CAD recommend a target value for LDL-C of 1450 (LDL-C <1.4 mmol l^−1^ or LDL-C reduced by more than 50%) to prevent coronary artery-related disease [9]. The proprotein convertase subtilisin/kexin type 9 (PCSK9) protease increases plasma LDL-C by decreasing LDL receptor (LDLR) expression on hepatocytes. Evolocumab, a human monoclonal immunoglobulin G2, specifically binds to human PCSK9 to lower LDL-C. In contrast to statins and ezetimibe, which act as feedback agents to upregulate LDLR, PCSK9 inhibitors (PCSK9i) directly upregulate LDLR levels by inhibiting PCSK9 [10]. The FOURIER-OLE study demonstrated that long-term use of evolocumab resulted in a significant, sustained and smooth decrease in lipids, improved achievement of plaque stabilization and even plaque reversal [11]. Recent work suggests pleiotropic effects of PCSK9i, including anti-inflammatory activity through SIRT3 upregulation [12]. Preclinical models reveal that gut microbiota modulates SIRT3 expression through butyrate-mediated histone deacetylation [13], suggesting potential bidirectional crosstalk between PCSK9 inhibition and microbial ecology.

In this prospective cohort study, 16S rRNA high-throughput sequencing was systematically employed to characterize gut microbial alterations associated with evolocumab therapy in 26 AMI patients. Our findings not only elucidate the pleiotropic cardioprotective mechanisms extending beyond lipid-lowering effects but also establish a novel conceptual framework for optimizing personalized therapeutic strategies in AMI management.

## Methods

### Study population

This prospective observational study enrolled 26 patients with AMI who were hospitalized in the Department of Cardiology, First Affiliated Hospital of Kunming Medical University, from September 2022 to August 2023, receiving statins. While not randomized, treatment allocation followed clinical guidelines. The AMI diagnosis of the patients was based on the World Health Organization definition and the third universal definition of myocardial infarction; these patients had symptoms of ischaemia, cardiac laboratory biomarker data and electrocardiogram results indicative of AMI and had undergone invasive coronary angiograms or coronary CT angiography [14]. The inclusion criteria were as follows: patients with a prior history of hyperlipidaemia who were treated with statins (rosuvastatin 10 mg qn or atorvastatin 20 mg qn) for at least 8 weeks before admission, patients whose ECG showed ST segment elevation or depression with elevated cTNI levels and patients who underwent emergency percutaneous coronary intervention (PCI) after admission. The exclusion criteria were as follows: (1) patients with heart failure or diabetes mellitus; (2) those who were pregnant or breastfeeding; (3) patients with a history of taking antibiotics and probiotics within 4 weeks; (4) those with gastrointestinal diseases and those who underwent gastrointestinal surgeries within 1 year; (5) patients with a history of acute gastroenteritis within the past week; (6) patients with a history of concomitant severe liver diseases, kidney diseases, autoimmune diseases, infectious diseases or malignant neoplasms; (7) those with cognitive and psychiatric disorders. Eighteen age-/sex-matched controls underwent identical screening. All participants provided written informed consent. Patients received subcutaneous evolocumab 420 mg every 4 weeks alongside statins. Faecal samples were collected at baseline (0W) and 8 weeks (8W). Enrolled patients underwent PCI and received guideline-directed secondary prevention medications. The distribution of baseline medications is summarized in Table 1. All patients received dual antiplatelet therapy (aspirin+P2Y12 inhibitors) and moderate-intensity statins (atorvastatin ≥20 mg day^−1^ or rosuvastatin ≥10 mg day^−1^). Other therapies included β-blockers (87.3%), ACEIs/ARBs (79.6%) and nitrates (62.1%). No dose adjustments or discontinuations of these medications were observed during the 8-week evolocumab treatment period.

**Table 1.** Baseline use of secondary prevention drugs (*n*=26)

Medication class	Patients receiving (%)	Dose range	Adjusted during 8 **W**
Aspirin	100%	81–100 mg day^−1^	No
P2Y12 inhibitors	100%	Clopidogrel 75 mg day^−1^	No
		Ticagrelor 90 mg BID	
Statins (moderate)	100%	Atorvastatin 20–40 mg	No
		Rosuvastatin 10–20 mg	
β-Blockers	87.3%	Metoprolol 50–200 mg	No
ACEIs/ARBs	79.6%	Lisinopril 5–40 mg	No
		Valsartan 80–320 mg	
Nitrates	62.1%	Isosorbide 20–60 mg BID	No

### Data collection

Demography, clinical data and medical history were collected on admission. The demographic data included age and sex, and the clinical data included body mass index, complications and medical history. Evaluation of blood samples before treatment with evolocumab (T0 W) and 8 weeks (T8 W) after treatment with evolocumab included routine blood tests and assessment of brain natriuretic peptide, myoglobin, creatine kinase-MB, troponin I and d-dimers. After fasting for 12 h, other blood samples were collected in strict accordance with standard procedures and sent to the laboratory of the First Affiliated Hospital of Kunming Medical University for immediate testing according to standard techniques. The indicators assessed included alanine aminotransferase, aspartate aminotransferase, creatinine, uric acid, fasting glucose, total cholesterol, triglycerides, LDL-C, high-density lipoprotein cholesterol, lipoprotein, white blood cells, haemoglobin and platelets.

### Collection of faecal samples

We collected fresh faecal samples from AMI patients and healthy controls. Fresh faecal samples were collected from all subjects on the second day after hospital admission, immediately placed in a liquid nitrogen tank and sent to the laboratory for storage at −80 °C. After 8 weeks of treatment with evolocumab in combination with statins, fresh stool samples were collected from the AMI-evolocumab (8 W) group. Fresh stool samples from all subjects were sent to Shanghai Meiji Biomedical Technology Co.

### Primary reagents and instruments

A TruSeq^™^ DNA Sample Prep Kit (Omega, USA), FastPfu Polymerase (Beijing All Style Gold Biotechnology Co., Ltd.), an ABI GeneAmp^®^ 9700 PCR instrument (ABI, USA) and a DYY-6C electrophoresis instrument (Beijing Liuyi Biotechnology Co., Ltd.) were used. High-throughput sequencing was performed by Shanghai Meiji Biomedical Technology Co.

### Methods

#### 16S RNA high-throughput sequencing

Faecal samples from the three groups of subjects were subjected to total genomic DNA extraction to obtain microbial DNA according to the instructions of the TruSeq^™^ DNA Sample Prep Kit (Omega Biotek, Georgia, USA). The integrity of the extracted genomic DNA was tested by agarose gel electrophoresis with 1% agarose, and the concentration and purity of the DNA were determined using a NanoDrop2000 (Thermo Fisher Scientific, USA). The extracted DNA was used as a template for PCR amplification of the V3-V4 variable region of the 16S rRNA gene using the upstream primer 338F (5′-ACTCCTACGGGGAGGCAGCAG-3′) and the downstream primer 806R (5′-GGACTACHVGGGTWTCTAAT-3′) [15], which carry a barcode sequence. PCR reactions contained 1X Phusion Master Mix, 0.2 µM primers and 10 ng DNA in 25 µl reactions. The amplification procedure was as follows: predenaturation at 95 °C for 3 min, 27 cycles of denaturation at 95 °C for 30 s, annealing at 55 °C for 30 s and extension at 72 °C for 30 s, followed by stable extension at 72 °C for 10 min, with final storage at 4 °C (PCR instrument: ABI GeneAmp9700). The PCR product was extracted from 2% agarose gel and purified using the PCR Clean-Up Kit (YuHua, Shanghai, China) according to the manufacturer’s instructions and quantified using Qubit 4.0 (Thermo Fisher Scientific, USA). Purified amplicons were pooled in equimolar amounts and paired-end sequenced on an Illumina Nextseq2000 platform (Illumina, San Diego, USA) according to the standard protocols by Majorbio Bio-Pharm Technology Co., Ltd. (Shanghai, China). The raw sequencing reads were deposited into the NCBI Sequence Read Archive database (accession number: PRJNA1154993). After demultiplexing, the resulting sequences were quality-filtered with fastp (0.19.6) [16] and merged with FLASH (v1.2.11) [17]. Then, the high-quality sequences were de-noised using the DADA2 [18] plugin in the Qiime2 [19] (version 2020.2) pipeline with recommended parameters, which obtains single-nucleotide resolution based on error profiles within samples. DADA2 denoised sequences are usually called amplicon sequence variants (ASVs). To minimize the effects of sequencing depth on alpha and beta diversity measures, the number of sequences from each sample was rarefied to 20,000, which still yielded an average Good’s coverage of 97.90%. Taxonomic assignment of ASVs was performed using the Naive Bayes consensus taxonomy classifier implemented in Qiime2 and the silva 16S rRNA database (v138) and then adjusted on the basis of the estimated rRNA operon copy number according to the *rrn*DB database [20].

#### Data analysis

All data analyses were performed on the Meggie BioCloud platform (https://cloud.majorbio.com) as follows: mothur [21] software (http://www.mothur.org/wiki/Calculators, version 1.30.2) was used to calculate the alpha diversity indices Chao and Shannon’s index; the Wilcoxon rank sum test was used for the analysis of between-group differences in alpha diversity, and bacterial taxa with significant differences in genus-level abundance between groups were analysed using the Kruskal–Wallis rank sum test. The *P*-values from Kruskal–Wallis H tests were adjusted for multiple comparisons using the Benjamini–Hochberg false discovery rate (FDR) correction. Differential abundance was assessed using ANCOM-BC to account for compositional data. *P-*values<0.05 were considered significant.

### Statistical analysis

Normally distributed data are expressed as the mean±sd, and between-group variability was tested using the t-test. Qualitative count data are expressed as percentages and were tested for between-group variability using the chi-square test. Nonnormally distributed data are expressed as medians and interquartile ranges (distances between the 25th and 75th percentiles) and were tested for differences using the Mann‒Whitney U test. All the data were analysed by two-tailed significance tests, with *P*<0.05 considered significant, using the statistical software SPSS (version 26.0).

## Results

### Characteristics of the study population

We recruited 26 patients (15 males and 11 females) with AMI to serve as the case group and 18 healthy individuals (11 males and 7 females) to serve as the control group. The baseline characteristics of the AMI patients and healthy controls were investigated after admission. No differences were observed in age, sex, body mass index, alanine aminotransferase, aspartate aminotransferase or fasting blood glucose between the AMI patients and the healthy controls (*P*>0.05). However, the level of cardiac troponin I and the incidence of hypertension were significantly greater in the patient group than in the control group (*P*<0.001). In addition, compared with those in the control group, the AMI group had significantly increased total cholesterol, triglyceride, LDL-C, lipoprotein (a), creatinine and uric acid and significantly decreased high-density lipoprotein cholesterol (*P*<0.05). White blood cell counts were greater in AMI patients than in healthy controls (*P*<0.001), but no difference was observed in haemoglobin or platelet counts between the AMI patients and the healthy controls (*P*>0.05), as shown in Table 2.

**Table 2.** Baseline characteristics of AMI patients and healthy controls

	Control (*N*=18)	AMI-evolocumab (0 W) (*N*=26)	*P*
Age (years)*	49.78±8.20	51.96±7.29	0.359
Male (n%)†	11 (61.1)	15 (57.7)	0.821
SBP (mmHg)*	111.56±4.03	133.50±21.08	<0.001
BMI (kg m^−2^)*	24.01±1.24	25.02±2.55	0.091
No.sv (n%)†			
1	na	4 (15.4)	
2	na	8 (30.8)	
3	na	14 (53.8)	
cTnI (ng ml^−1^)‡	0.00 (0.00, 0.00)	7.05 (3.80, 17.10)	<0.001
Hypertension (n%)†	0 (0)	16 (61.5)	<0.001
ALT (IU l^−1^)‡	19.65 (15.80, 24.80)	24.95 (20.30, 33.35)	0.358
AST (IU l^−1^)‡	20.80 (16.58, 24.80)	22.10 (16.80, 32.15)	0.995
Creatinine (μmol l^−1^)*	61.28±9.15	76.54±13.05	<0.001
Uric acid (μmol l^−1^)*	263.14±54.06	333.76±73.37	0.001
Glucose (mmol l^−1^)*	4.97±0.39	4.83±0.47	0.334
TC (mmol l^−1^)*	4.01±0.50	4.85±0.94	<0.001
TG (mmol l^−1^)*	1.44±0.39	2.03±0.95	0.007
HDL-C (mmol l^−1^)*	1.70±0.23	1.09±0.27	<0.001
LDL-C (mmol l^−1^)*	2.19±0.48	3.57±0.80	<0.001
Lpa (mmol l^−1^)*	36.71±16.82	197.75±151.00	<0.001
WBC (×10⁹ L⁻¹)*	6.38±1.37	9.69±2.94	<0.001
Hb (g l^−1^)*	150.28±8.50	156.38±12.92	0.086
PLT (×10⁹ L⁻¹)*	228.17±46.52	230.19±48.19	0.89

*Mean±sd. †n (%). ‡Median (IQR).

Continuous variables are presented as mean±sd if normally distributed or median [interquartile range (IQR)] if non-normally distributed. Categorical variables are expressed as number (%).

To compare the differences in clinical characteristics between the two groups, independent sample t tests were used for continuous normally distributed data. The Mann‒Whitney U test was used for continuous data that were not normally distributed between the two groups, and categorical variables were compared by chi-square tests. Normality was assessed using the Shapiro–Wilk test, and homogeneity of variance was confirmed via Levene’s test. A *P-*value <0.05 indicated statistical significance.

### Changes in the biochemical indices of AMI patients after 8 weeks of evolocumab treatment

After treatment with evolocumab, the total cholesterol, triglyceride, LDL-C and lipoprotein(a) levels in AMI patients were significantly decreased (*P*<0.01), while the high-density lipoprotein cholesterol level was significantly increased (*P*<0.001). In addition, the levels of white blood cells, creatinine and uric acid were markedly reduced compared to baseline measurements (*P*<0.001). However, no significant differences were found in fasting blood glucose, alanine aminotransferase, aspartate aminotransferase, haemoglobin or platelet levels (*P*>0.05), as shown in Table 3.

**Table 3.** Changes in blood biochemistry and inflammation after 8 weeks of evolocumab treatment

	AMI-evolocumab (0 W)	AMI-evolocumab (8 W)	*P*
ALT (IU l^−1^)‡	24.95 (20.30, 33.35)	20.6 (17.38, 25.90)	0.052
AST (IU l^−1^)‡	22.10 (16.80, 32.15)	20.15 (15.75, 25.78)	0.267
Creatinine (μmol l^−1^)*	76.54±13.05	66.71±11.66	<0.001
Uric acid (μmol l^−1^)*	333.76±73.37	269.93±58.04	<0.001
Glucose (mmol l^−1^)*	4.83±0.47	4.75±0.40	0.289
TC (mmol l^−1^)*	4.85±0.94	2.12±0.51	<0.001
TG (mmol l^−1^)*	2.03±0.95	1.08±0.34	<0.001
HDL-C (mmol l^−1^)*	1.09±0.27	1.42±0.32	<0.001
LDL-C (mmol l^−1^)*	3.57±0.80	0.83±0.33	<0.001
Lpa (mmol l^−1^)*	197.75±151.00	75.67±90.32	<0.001
WBC (×10⁹ L⁻¹)*	9.69±2.94	6.12±1.55	<0.001
Hb (g l^−1^)*	156.38±12.92	154.65±9.78	0.655
PLT (×10⁹ L⁻¹)*	230.19±48.19	226.85±40.81	

*Mean±sd. †n (%). ‡Median (IQR).

Continuous variables are presented as mean±sd if normally distributed or median [interquartile range (IQR)] if non-normally distributed. Categorical variables are expressed as number (%).

Paired sample t tests and Wilcoxon signed-rank tests were used. Normality was assessed using the Shapiro–Wilk test, and homogeneity of variance was confirmed via Levene’s test. A *P-*value <0.05 indicated statistical significance.

### Taxonomic diversity profiling and community structure analysis of gut microbiota

**Fig. 1.**
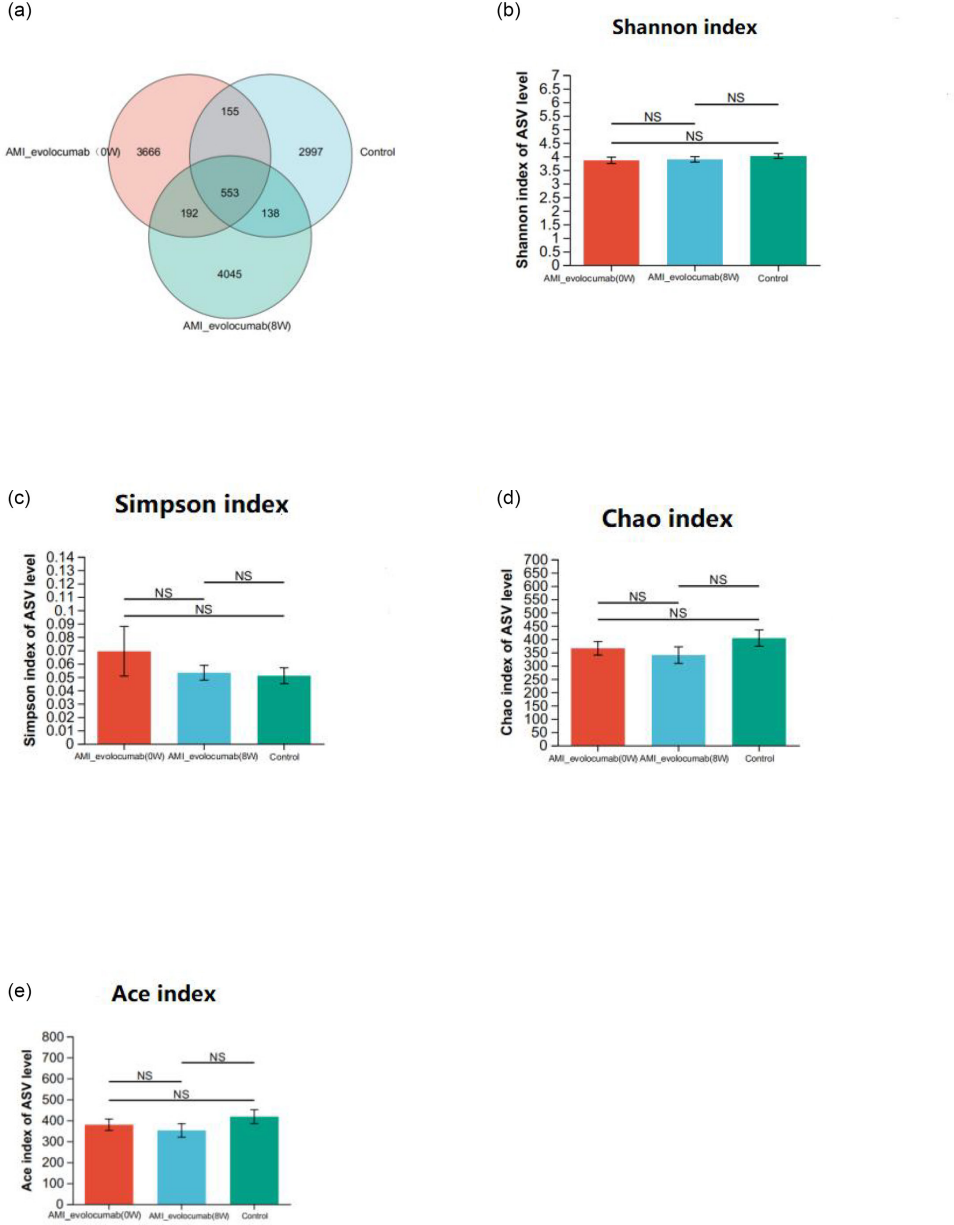
Characteristics of faecal microbiota and alpha diversity analysis. (**a**) A Venn diagram showing the overlap of ASVs shared between the conditions. The bar diagram of Shannon index (**b**), Simpson index (**c**), Chao index (**d**) and Ace index (**e**) was shown to analyse microbiome diversity. NS: not significant, Kruskal–Wallis H test.

An average of 11,540 high-quality sequences were obtained per faecal sample by high-throughput 16S rRNA sequencing. A Venn diagram was constructed based on the ASV clustering results (Fig. 1a). In all, 553 ASVs were shared by the three groups, and 192 ASVs were common to the two disease clusters. In addition, 138 ASVs were shared by the control and AMI-evolocumab (0 W) groups, and 155 ASVs were shared by the control and AMI-evolocumab (8 W) groups. In terms of alpha diversity, we observed no significant differences in the Shannon, Simpson, Chao or Ace indices among the three groups (Fig. 1b–e), which indicates no significant differences in the overall structure of the intestinal flora among the three groups (*P*>0.05).

### Overview of the gut microbiota at the phylum and genus levels

**Fig. 2.**
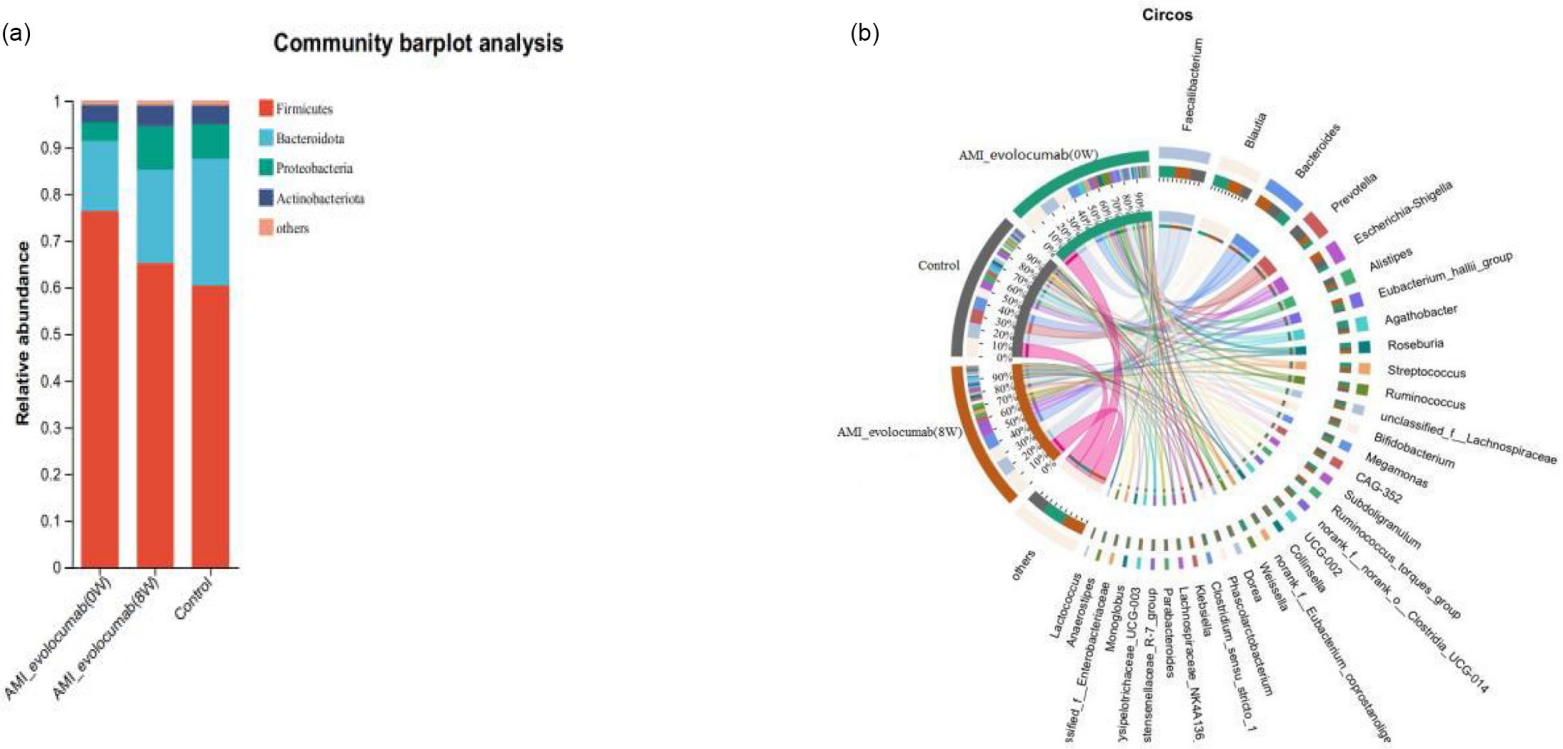
Overview of the gut microbiome in different groups. (**a**) Dominant phyla in each group. (**b**) Dominant genera and their contribution to each group.

At the phylum level, four bacterial taxa consistently dominated all cohorts (control, AMI-evolocumab 0 W and 8 W), collectively accounting for >99% of total sequences (Fig. 2a). The most dominant phylum was *Firmicutes*, with a relative abundance of 76.32% in the AMI-evolocumab (0 W) group, 65.22% in the AMI-evolocumab (8 W) group and 60.40% in the control group. The second most dominant phylum was *Bacteroidota* (control: 27.11%; AMI-evolocumab (0 W): 15.07%; AMI-evolocumab (8 W): 19.99%). *Proteobacteria* and *Actinobacteria* phyla were also detected. The most abundant microbes at the genus level and their contributions to each taxon are shown in Fig. 2(b), with *Faecalibacterium*, *Blautia*, *Bacteroides* and *Prevotella* being the most dominant genera.

### Alterations in the gut microbiota composition associated with evolocumab use

**Fig. 3.**
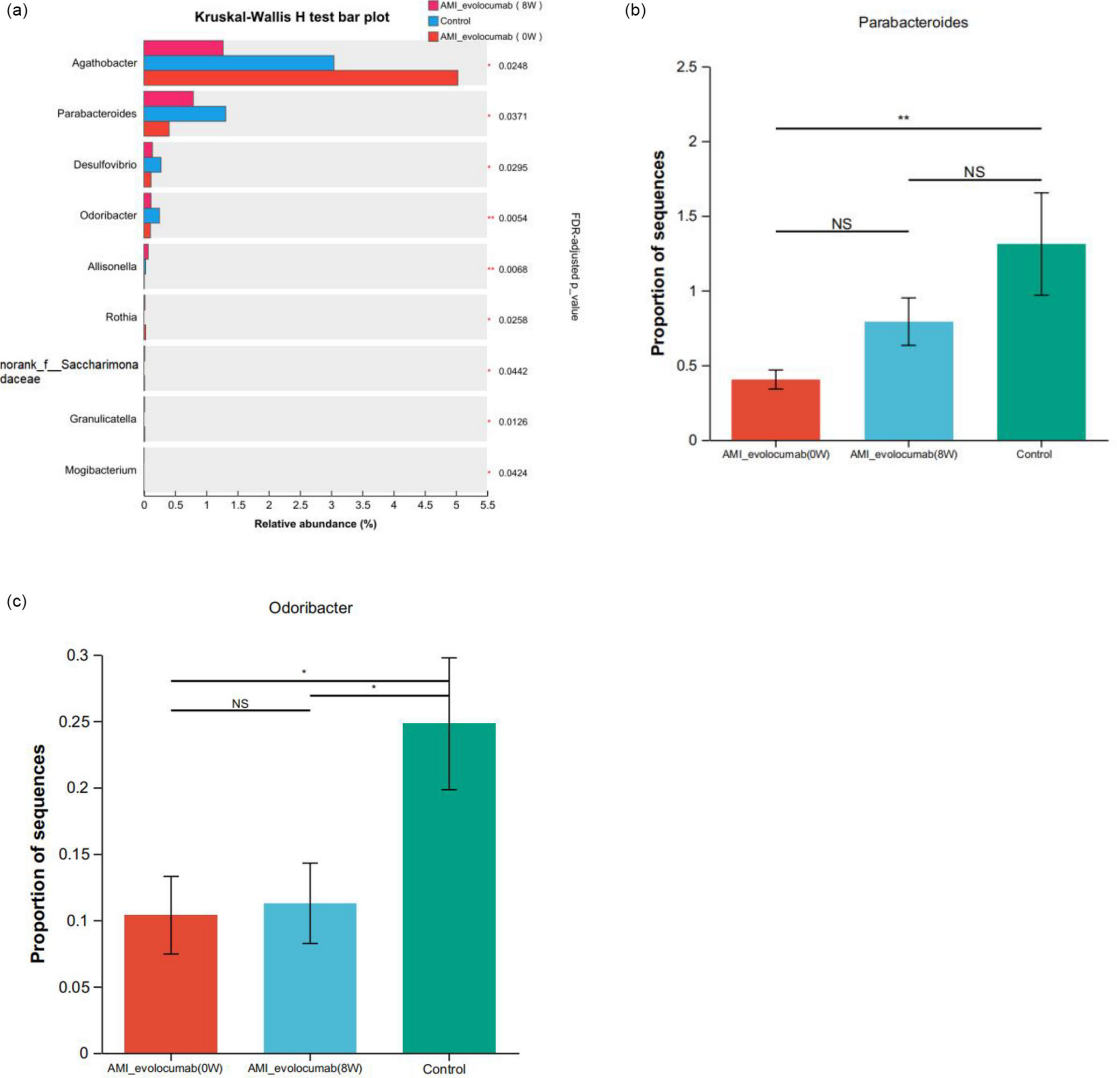
Differential abundance analysis of gut microbiota at the genus level across three groups. (**a**) Kruskal–Wallis H tests with Benjamini–Hochberg FDR correction were performed for comparisons. Significantly different genera (FDR-adjusted *P*<0.05) are labelled with asterisks: *P<0.05, **P<0.01. (**b, c**) Two-by-two comparison results between the three groups. Post-hoc test. *P<0.05, **P<0.01. A *P*-value of <0.05 was considered statistically significant.

Comparative analysis revealed significant intergroup variations in the relative abundances of *Firmicutes* and *Bacteroidota*. The abundance of the *Firmicutes* phylum in the AMI-evolocumab (0 W) group was significantly greater than that in the AMI-evolocumab (8 W) group and the control group (*P*<0.01). The abundance of *Bacteroidota* in the AMI-evolocumab (0 W) group was significantly lower than that in the control group (*P*<0.01), but no significant difference was observed between the AMI-evolocumab (8 W) group and the control group (*P*>0.05). No significant difference was found in the abundance of *Proteobacteria* or *Actinobacteria* among the three groups (*P*>0.05). Kruskal–Wallis testing with Benjamini–Hochberg FDR correction identified nine genera showing significant intergroup variation (FDR-adjusted *P*<0.05): *Agathobacter*, *Parabacteroides*, *Desulfovibrio*, *Odoribacter*, *Allisonella*, *Rothia*, *Granulicatella*, *Mogibacterium* and *norank_f__Saccharimonadaceae* (family-level taxon retained due to unresolved genus classification in GTDB r214) (Fig. 3a). In the pairwise comparative analyses (Fig. 3b, c), no statistically significant differences were observed in the abundance of bacterial genera except for *Parabacteroides* and *Odoribacter*. Specifically, the abundance of *Parabacteroides* was significantly higher in the control group than in the AMI-evolocumab (0 W) group (*P*<0.05) but showed no significant difference compared to the AMI-evolocumab (8 W) group (*P*>0.05). Furthermore, the abundance of *Odoribacter* in the control group was significantly greater than that in both the AMI-evolocumab (0 W) and AMI-evolocumab (8 W) groups (*P*<0.05). In contrast, no statistically significant differences were observed in the abundance of any other bacterial genera across the groups (*P*>0.05), indicating that the alterations in microbial community structure were highly genus-specific, with only *Parabacteroides* and *Odoribacter* exhibiting significant responses to the intervention or disease state. Overall, the gut microbial structure of the AMI-evolocumab (8 W) group was more like that of the healthy control group, which suggests a relationship between the relative composition of the microbiome and treatment with evolocumab.

## Discussion

5The study by Han *et al*. [22] showed that compared with healthy controls, AMI patients had a lower abundance of *Firmicutes* and a slightly greater abundance of *Bacteroidetes* in their intestinal flora. In terms of genera, the abundances of *Megasphaera*, *Butyricimonas*, *Acidaminococcus* and *Desulfovibrio* are relatively high in AMI patients. The abundances of *Tyzzerella 3*, *Dialister*, *Eubacterium ventriosum group*, *Pseudobutyrivibrio* and *Lachnospiraceae ND3007 group* were low. The study by Wu *et al*. [23] showed that after AMI, the intestinal flora of mice exhibited increases in *Firmicutes* and decreases in *Bacteroidetes*. After AMI, the abundances of some pathogenic bacteria increased, such as those of the *Syntrophomonadaceae* family, the *Eubacteriaceae* family, the *Tissierella* genus and the *Soehngenia* genus. The genera *Tissierella* and *Soehngenia* belong to the phylum *Firmicutes*. A possible explanation for these findings is that differences in the gut environment may affect the abundance and composition of the gut microbiota. In this study, we compared the differences in the faecal microbiota among three groups of subjects using 16S rRNA gene sequencing. From a macro perspective, although α-diversity analysis revealed no significant differences in overall gut bacterial richness among the three groups, distinct differences in the relative abundance of specific bacterial phyla were observed. Significant differences in the relative abundances of *Firmicutes* and *Bacteroidetes* phyla were observed between AMI patients receiving long-term statin therapy (>8 weeks) and healthy controls, whereas alterations in other phyla showed less pronounced abundance variations. Compared with that in the control group, the proportion of *Firmicutes* in the AMI-evolocumab (0 W) group was increased, while the proportion of *Bacteroides* was decreased, the difference of which was statistically significant, which was similar to the trend demonstrated in a case‒control study of CAD [24]. In a comparison of two patient groups with different statin efficacies, the genera *Akkermansia muciniphila* and *Lactobacillus*, which are beneficial for lipid metabolism, were found at lower abundances in those with poor statin efficacy, whereas the genera *Holdemanella* and *Faecalibacterium* were found at higher abundances [25], which suggests an interaction between the gut flora and drug therapy. In addition, evidence suggests that statins may confer protective effects in patients with CAD, potentially through direct or indirect modulation of gut microbiota composition and function [26]. After 8 weeks of treatment with the three-injection regimen of evolocumab, the proportion of *Firmicutes* in AMI patients decreased, while the proportion of Bacteroides increased. As a result, we found no significant difference in the abundance of the phyla *Firmicutes* and Bacteroides between the AMI-evolocumab (8 W) group and the control group. In addition, compared with that in the control group, the *Firmicutes*/*Bacteroidetes* ratio in the AMI-evolocumab (0 W) group was increased, while the *Firmicutes*/*Bacteroidetes* ratio was decreased after evolocumab treatment. Previous studies have shown that an increase in the *Firmicutes*/*Bacteroidetes* ratio is positively correlated with obesity and dyslipidaemia [27]. Therefore, our findings suggest that evolocumab treatment in AMI patients is associated with shifts in gut microbiota structure, characterized by a reduction in potential pathogenic bacteria and an increase in beneficial taxa. Whether they represent a direct mechanistic effect of evolocumab or an indirect consequence of its lipid-lowering action remains unclear. Based on the observed parallels with statin-associated microbiota modulation, we hypothesize that evolocumab may share pleiotropic properties that extend beyond lipid metabolism, potentially contributing to a more favourable gut microbial environment. However, further studies are required to dissect causality and determine whether microbiota alterations are drivers or bystanders of therapeutic benefits.

The genera with the highest abundances were *Faecalibacterium*, *Blautia*, *Bacteroides* and *Prevotella*. The genus *Faecalibacterium* is an important probiotic in the human body and plays important roles in immune system regulation, intestinal barrier protection and microbial community regulation. In addition, bacteria of this genus have been shown to produce butyrate and anti-inflammatory effects [28]. Butyric acid is a short-chain fatty acid that can maintain the integrity of the intestinal barrier, enter the blood through intestinal mucosal epithelial monocarboxylic transporters and regulate liver lipid metabolism through the G protein-coupled receptors GPR41 and GPR43 [29]. While our study revealed no significant differences in *Faecalibacterium* abundance among the three groups, this stability may reflect its essential role in gut homeostasis, which could be preserved – rather than directly modulated – by therapeutic interventions such as statins or evolocumab. The proportion of *Bacteroides* in the three groups was 28% in the AMI-evolocumab (0 W) group, 39% in the AMI-evolocumab (8 W) group and 34% in the control group. Although the difference was not statistically significant, the abundance of *Bacteroides* in the AMI-evolocumab (8 W) group was increased. *Bacteroides* is also an important probiotic in the human body that participates in carbohydrate fermentation and the metabolism of polysaccharides, bile acids and steroids and has an important impact on human health. A decrease in *Bacteroides* leads to increased susceptibility to the accumulation of fat and an increase in intestinal lipoprotein, which leads to an increase in inflammatory reactions, insulin resistance and other coronary heart disease risk factors [30].

By analysing differences between groups, we found nine species with differences at the genus level among the three groups. The abundance of *Parabacteroides* in the AMI-evolocumab (0 W) group was significantly lower than that in the control group, but no significant difference was observed between the AMI-evolocumab (8 W) group and the control group. The abundance of *Odoribacter* in the control group was significantly greater than that in the AMI-evolocumab (0 W) group and the AMI-evolocumab (8 W) group. Microbes of the *Parabacteroides* genus possess the physiological abilities to participate in carbohydrate metabolism and the secretion of short-chain fatty acids. In addition, microbes in the *Parabacteroides* genus exert protective effects against inflammation and obesity in mice [31]. A decrease in the abundance of *Odoribacter* may reduce SCFA production, thereby diminishing anti-inflammatory effects and potentially contributing to increased intestinal inflammation [32]. In addition, the presence of *Odoribacter* is beneficial to healthy systolic blood pressure [33]. The gut microbiota of AMI patients receiving evolocumab showed partial overlap with both untreated AMI patients and healthy controls. Notably, after 8 weeks of evolocumab therapy, the microbial composition shifted toward a profile more closely resembling that of healthy individuals compared to baseline (0 W). While our data suggest that evolocumab may modulate gut microbiota composition independently of statin-like effects, it is noteworthy that certain statins themselves have been associated with reduced dysbiosis [34]. This highlights a potential complementary role of lipid-lowering therapies in microbiome homeostasis, warranting further comparative studies.

The intestinal microbial ecosystem is recognized as a necessary endocrine organ that can produce a variety of bioactive compounds, which can be released into the peripheral circulation, where they interact with multiple organs of the host. Our study provides insight into the changes in intestinal micro-organisms that occur in patients with AMI after evolocumab treatment. However, this study has several limitations. We investigated only microbial characteristics and did not study changes in serum metabolites; thus, we could not analyse how evolocumab benefits the host by regulating the intestinal microflora. Since the human intestinal microecology is quite complex, evolocumab may regulate intestinal micro-organisms through complex channels rather than by acting directly on bacteria. In addition, the results of this study are influenced by many confounding factors (e.g. treatment with drugs other than evolocumab and statins, diet and lifestyle). In addition, due to the relatively small sample size, data interpretation may be limited, and our conclusion requires verification by larger-scale research. Nevertheless, our study showed that evolocumab can regulate the intestinal microflora, which may explain the protective effect of evolocumab on AMI, although further verification and exploration through metabolic and functional studies are needed.

## Conclusion

We speculate that evolocumab, the first-line drug for lipid-lowering treatment of coronary heart disease, has the same pleiotropic effect as statins. Evolocumab may benefit AMI patients by regulating their intestinal flora to elicit a healthier state; that is, the drug may reduce potential pathogenic bacteria and increase beneficial bacteria, thus reducing the metabolic risk of these patients. Our study provides new insights into the mechanism of action of evolocumab in the treatment of AMI patients. Evolocumab demonstrated cardiovascular protection in AMI in a heterogeneous manner, although further exploration and verification in metabolic and functional studies are needed.

## Supplementary material

10.1099/jmm.0.002114 Uncited Supplementary Material 1.

## Abbreviations

AMI: acute myocardial infarction
ASVs: amplicon sequence variants
CRP: C-reactive Protein
FDR: false discovery rate
LDL-C: low-density lipoprotein cholesterol
LDLR: LDL receptor
PCI: percutaneous coronary intervention
PCSK9: proprotein convertase subtilisin/kexin type 9
PCSK9i: PCSK9 inhibitors
TMAO: trimethylamine N-oxide
